# Human Pluripotent Stem Cells Differentiated in Fully Defined Medium Generate Hematopoietic CD34^+^ and CD34^−^ Progenitors with Distinct Characteristics

**DOI:** 10.1371/journal.pone.0014733

**Published:** 2011-02-25

**Authors:** Laurie Chicha, Anis Feki, Alessandro Boni, Olivier Irion, Outi Hovatta, Marisa Jaconi

**Affiliations:** 1 Department of Pathology and Immunology, Faculty of Medicine, Geneva University, Geneva, Switzerland; 2 Stem Cell Research Laboratory, Department of Gynecology and Obstetrics, Geneva University Hospital, Geneva, Switzerland; 3 Department of Clinical Science, Intervention and Technology, Karolinska Institutet, Stockholm, Sweden; Brigham and Women's Hospital, United States of America

## Abstract

**Background:**

Differentiation of pluripotent stem cells *in vitro* provides a powerful means to investigate early developmental fates, including hematopoiesis. In particular, the use of a fully defined medium (FDM) would avoid biases induced by unidentified factors contained in serum, and would also allow key molecular mediators involved in such a process to be identified. Our goal was to induce *in vitro*, the differentiation of human embryonic stem cells (ESC) and induced pluripotent stem cells (iPSC) into morphologically and phenotypically mature leukocytes and erythrocytes, in the complete absence of serum and feeder cells.

**Methodology/Principal Findings:**

ESC and iPSC were sequentially induced in liquid cultures for 4 days with bone morphogenic protein-4, and for 4 days with FLT3-ligand, stem cell factor, thrombopoietin and vascular endothelium growth factor. Cell differentiation status was investigated by both mRNA expression and FACS expression profiles. Cells were further sorted and assayed for their hematopoietic properties in colony-forming unit (CFU) assays. In liquid cultures, cells progressively down-modulated Oct-4 expression while a sizeable cell fraction expressed CD34 *de novo*. SCL/Tal1 and Runx1 transcripts were exclusively detected in CD34^+^ cells. In clonal assays, both ESC and iPSC-derived cells generated CFU, albeit with a 150-fold lower efficacy than cord blood (CB) CD34^+^ cells. ESC-derived CD34^+^ cells generated myeloid and fully hemoglobinized erythroid cells whereas CD34^−^ cells almost exclusively generated small erythroid colonies. Both ESC and iPSC-derived erythroid cells expressed embryonic and fetal globins but were unable to synthesize adult β-globin in contrast with CB cells, suggesting that they had differentiated from primitive rather than from definitive hematopoietic progenitors.

**Conclusions/Significance:**

Short-term, animal protein-free culture conditions are sufficient to sustain the differentiation of human ESC and iPSC into primitive hematopoietic progenitors, which, in turn, produce more mature blood cell types. However, additional factors have yet to be identified to allow their differentiation into definitive erythroid cultures.

## Introduction

Embryonic stem cells (ESC) are derived from the inner cell mass of the pre-implantation blastocyst [1]. These cells are pluripotent, self-renewing, and can differentiate into all three embryonic germ layers – ectoderm, endoderm and mesoderm. Several studies have already identified defined culture conditions for directing ESC differentiation toward the mesodermal hematopoietic lineage [2], [3], [4], [5], [6], and more specifically, terminally differentiated erythrocytes and leukocytes have been generated through these strategies [2], [3], [7], [8], [9]. These studies however, used mouse embryonic fibroblasts as feeder support and poorly defined xenogenic compounds including serum prior to lineage-specific differentiation. As serum is replete with various growth factors and growth factor precursors, such an approach may lead to inter-species contamination, a fact that would preclude the use of the generated cells for clinical application. Moreover, the use of ESC raises both immunological [10], [11] and, more controversially, ethical concerns that may also preclude their use as therapeutic tools. The process of somatic cell reprogramming, which allows the generation of pluripotent stem cells exhibiting similar properties to ESC, may help bypass these concerns [12], [13], [14]. Recently, two studies showed that human iPSC could be differentiated toward hematopoiesis [15], [16], however, as for earlier studies with ESC, experiments were performed in bovine serum-supplemented cocultures, in the presence of murine stroma. Thus, it remains to be established whether xenogenic compounds are dispensable for the differentiation of human ESC and iPSC toward the hematopoietic lineage.

Here we show that human ESC and iPSC maintained over mitotically-inactivated human foreskin fibroblasts (HFF) [14] can differentiate toward the hematopoietic lineage in fully defined medium devoid of animal proteins when sequentially exposed to recombinant bone morphogenic protein-4 (BMP-4), a factor known to enhance mesodermal differentiation of human ESC *in vitro* [17], [18], [19], and to a cocktail of early acting hematopoietic factors comprising FMS-like tyrosin kinase-3 ligand (FLT3-L), stem cell factor (SCF), thrombopoietin (TPO) and vascular endothelial growth factor (VEGF). Within 8 days, cells down-modulated Oct-4 expression and upregulated hematopoietic transcription factors such as SCL/Tal1 and Runx1. Cell suspensions contained hematopoietic progenitors generating erythroid and granulocyte-macrophage colonies in methylcellulose. It appeared; however, that ESC- and iPSC-derived hematopoietic cells remained at an early stage of differentiation, corresponding to transient, primitive hematopoiesis, as they only expressed embryonic and fetal globins, but not adult β-globins, which was in contrast with CB cells.

## Results

### Differentiation of embryonic stem cells (ESC) toward the hematopoietic lineage

**Figure 1:**
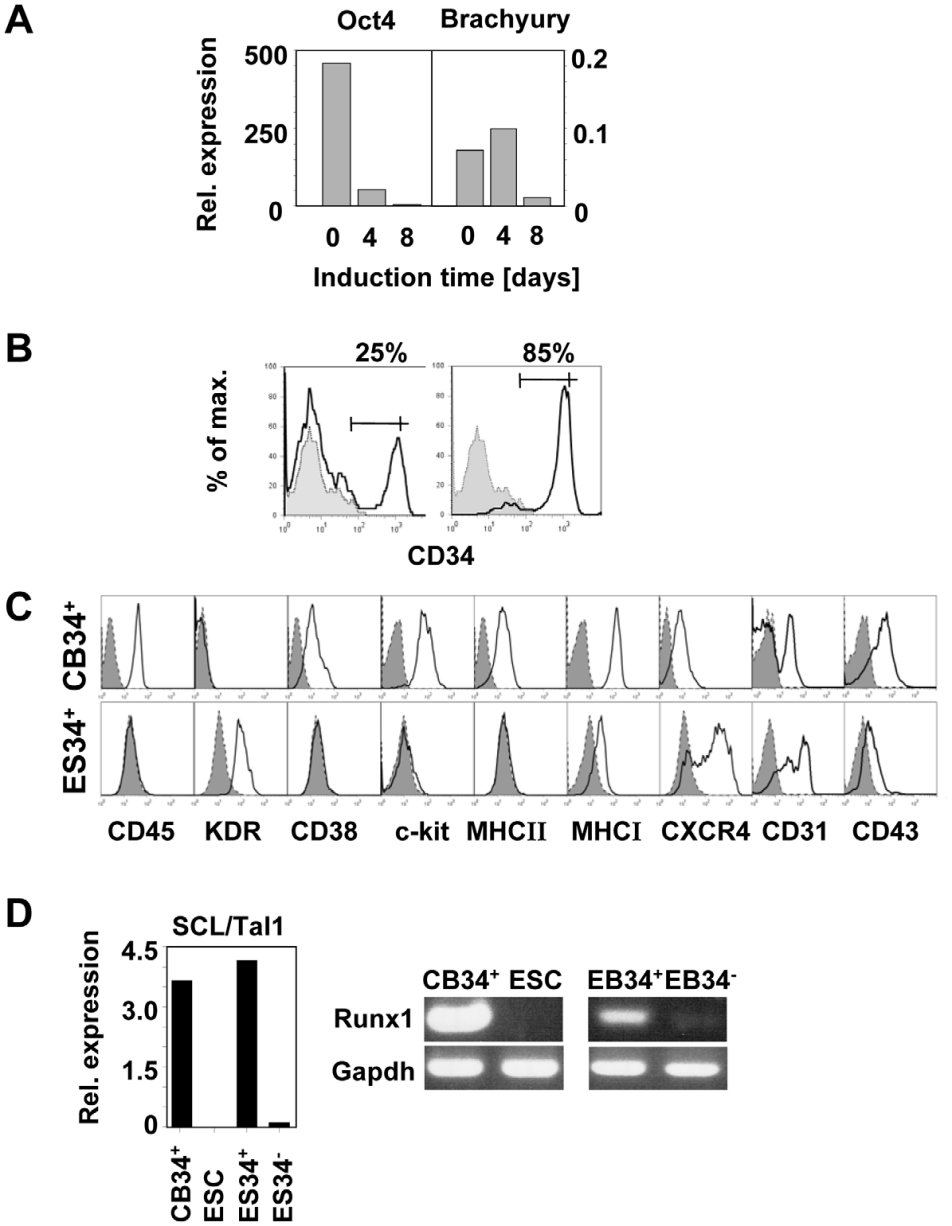
Modification of ESC gene and surface marker expression during in vitro differentiation in fully defined medium. (A) Oct-4 and Brachyury mRNA levels were assessed by quantitative RT-PCR at the onset of the differentiation (d0) and 4 (d4) and 8 (d8) days later. Data were normalized with Gus-B housekeeping gene expression (Representative data of 4 independent experiments). (B) CD34 expression was assessed by flow cytometry upon 8 days of differentiation before (black line, left panel) and after (black line, right panel) MACS selection. Grey profiles represent CD34 expression before hematopoietic differentiation. (C) Analysis of relevant markers was performed on MACS-enriched ES34^+^ after 8 days of differentiation and compared to CB34^+^ sorted cells. (D) SCL/Tal1 and Runx1 mRNA levels on indicated populations were assessed by quantitative RT-PCR and RT-PCR, respectively. Data are representative of 4 independent experiments.

During the initial 4-day induction phase with BMP-4 and bFGF in hypoxic conditions, ESC differentiated toward early mesodermal progenitors, as seen by the rapid decrease of Oct-4 expression and the transient induction of Brachyury at day 4 (**Fig. 1A**). After a subsequent induction of 4 days with VEGF, bFGF, SCF, FLT3-L and TPO in the absence of BMP-4, Oct-4 expression vanished, Brachyury sharply decreased and CD34^+^ cells, undetectable in undifferentiated ESC suspensions, reached 16±6% (mean±SD, n = 9, **Fig. 1B** left panel). Differentiated ESC were sorted by MACS according to CD34 expression. Mean purity was 73±17% (mean±SD, n = 8, **Fig. 1B** right panel) for the CD34-enriched fraction (defined as ES34^+^) and no contaminating CD34^+^ cells were detected in the ES34^−^ fractions upon reanalysis (not shown). FACS analysis of ES34^+^ demonstrated that they were CD45-, MHC-II- and CD38-negative, expressed low levels of MHC-I, CD43 and c-kit (CD117), and were positive for KDR, CXCR4 and CD31. This phenotype, though distinct from *ex vivo* CB-purified CD34^+^ cells (CB34^+^, **Fig. 1C**) may be consistent with very immature hematopoietic stem cells (HSC), likely sharing properties with the hemangioblast [4], [17], [18]. mRNA analysis performed on ES34^+^ and ES34^−^ fractions showed that SCL/Tal1, which exerts its effect immediately downstream of Brachyury to differentiate hemangioblasts into haemogenic endothelium [20], and Runx1, which is mainly expressed in cells differentiating further from this later step to the hematopoietic lineage [20], were exclusively expressed in ES34^+^ (**Fig. 1D**). Altogether, these observations suggest that our protocol generates a significant number of cells with a phenotype reminiscent of hematopoietic progenitors, yet in an immature stage of differentiation.

### ES34^+^ hematopoietic progeny generated *in vitro* is larger and contains more myeloid cells than that of ES34^−^

**Figure 2:**
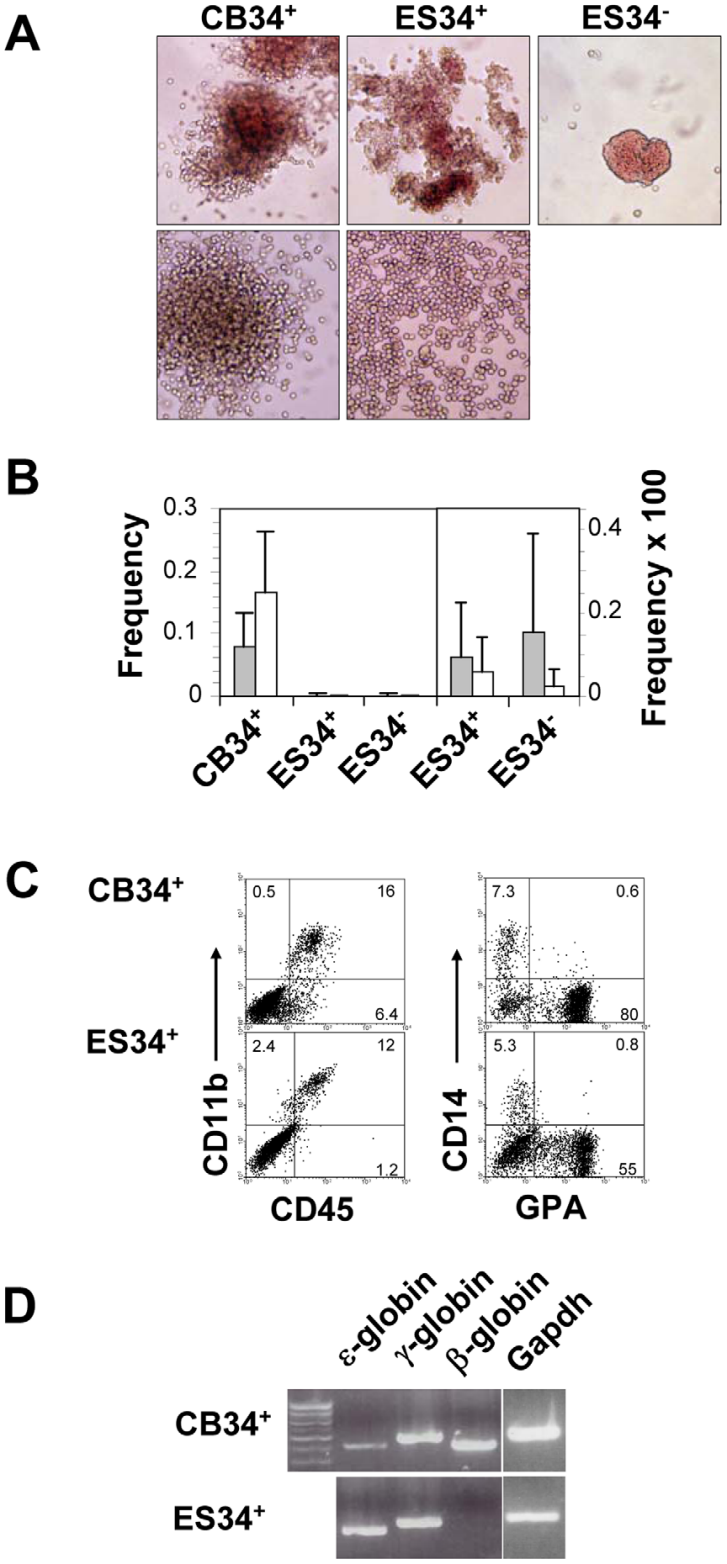
Cells differentiated from ESC are functional hematopoietic progenitors. (A) Colony morphology upon 14 days of methylcellulose culture. Erythroid CFU (upper panels) and myeloid CFU (lower panels) are shown (initial magnification ×100). (B) CFU frequencies of CB34^+^, ES34^+^ and ES34^−^. Overall frequencies (left panel) of CB and ESC-derived cells, and a close-up of ES34^+^ and ES34^−^ frequencies (right panel) displayed with an enlarged scale to help reading are depicted. Grey columns represent erythroids and white columns, myeloids. Bars are SD's. n = 8, 12 and 5 for CB, ES34^+^ and ES34^−^ respectively. (C) FACS analysis of cells recovered after the CFU assay derived from CB34^+^ (upper panels) and ES34^+^ (lower panels). Myeloid (CD45, CD11b, CD14) and erythroid (GPA) cell surface markers are depicted. Quadrants were established using appropriate isotype controls and percentages are indicated. (D) Qualitative analysis of embryonic (ε), fetal (γ) and adult (β) globin mRNAs expression by RT-PCR of cell suspensions recovered after the CFU assays, derived from the indicated populations.

ES34^+^ and ES34^−^ were tested for their ability to proliferate clonally in methylcellulose-based medium complemented with hematopoietic growth factors. After 14 days of culture, both cell suspensions generated colonies. Myeloid (CFU-GM) and erythroid colonies (BFU-E) derived from ES34^+^ were morphologically similar to hematopoietic colonies derived from CB34^+^ cells. By contrast ES34^−^ produced almost exclusively small-sized erythroid colonies with a limited burst promoting activity which looked more like CFU-E rather than BFU-E. Myeloid colonies derived from ES34^−^ were scarce and could not be documented by photographic means (**Fig. 2A**). The frequency of hematopoietic colonies obtained from ESC-generated hematopoietic progenitors was approximately 160-fold lower than those obtained from CB34^+^ cells (**Fig. 2B**, left panel). Erythroid and CFU-GM colony frequencies were 9.7×10^−4^ and 6.1×10^−4^ for ES34^+^ (n = 12) and 1.6×10^−3^ and 2.5×10^−4^ for ES34^−^ (n = 5) respectively (**Fig. 2B**, right panel). Such a low cloning efficiency led to a large dispersion of the values that did not allow establishing a significant difference between ES34^+^ and ES34^−^. The erythroid/myeloid colony ratio was 34% for CB34^+^, 61% for ES34^+^ and 86% for ES34^−^ cells. Colonies derived from CB34^+^ contained in average 2.1×10^4^ cells/colony, whereas colonies obtained from ES34^+^ or ES34^−^ cells contained 3×10^3^ and 1.7×10^3^ cells/colony, respectively. Thus, ESC differentiated in our assay generated both myeloid and erythroid colonies, albeit at a lower efficiency as compared with CB.

Cell suspensions derived from the clonal cultures of ES34^+^ were then analyzed by FACS (ES34^−^ numbers were not sufficient to allow for FACS analysis) and compared to the progeny of CB34^+^ cells. Cultures derived from ES34^+^ cells contained both myeloid and erythroid progenitors (**Fig. 2C**), and, as for CB-derived cultures, CD34^+^ cells were no more detectable (data not shown), consistent with the differentiation of the progenitors observed in CFU assays. Cells were also tested for globin gene expression. While CB-derived CFU expressed embryonic (ε), fetal (γ) and adult (β) globin mRNAs, ES34^+^ exclusively expressed ε and γ globins after *in vitro* differentiation (**Fig. 2D**). Altogether these observations - i.e. low frequency of hematopoietic colonies, limited proliferation, bias toward erythropoiesis and absence of adult globin synthesis - suggest that ESC differentiated *in vitro* exhibit a block in further differentiation that prevents them to reach the same stage than CB-HSC.

### Reprogrammed human somatic cells (iPSC) also generate hematopoietic progenitors in animal protein-free differentiation cultures

**Figure 3:**
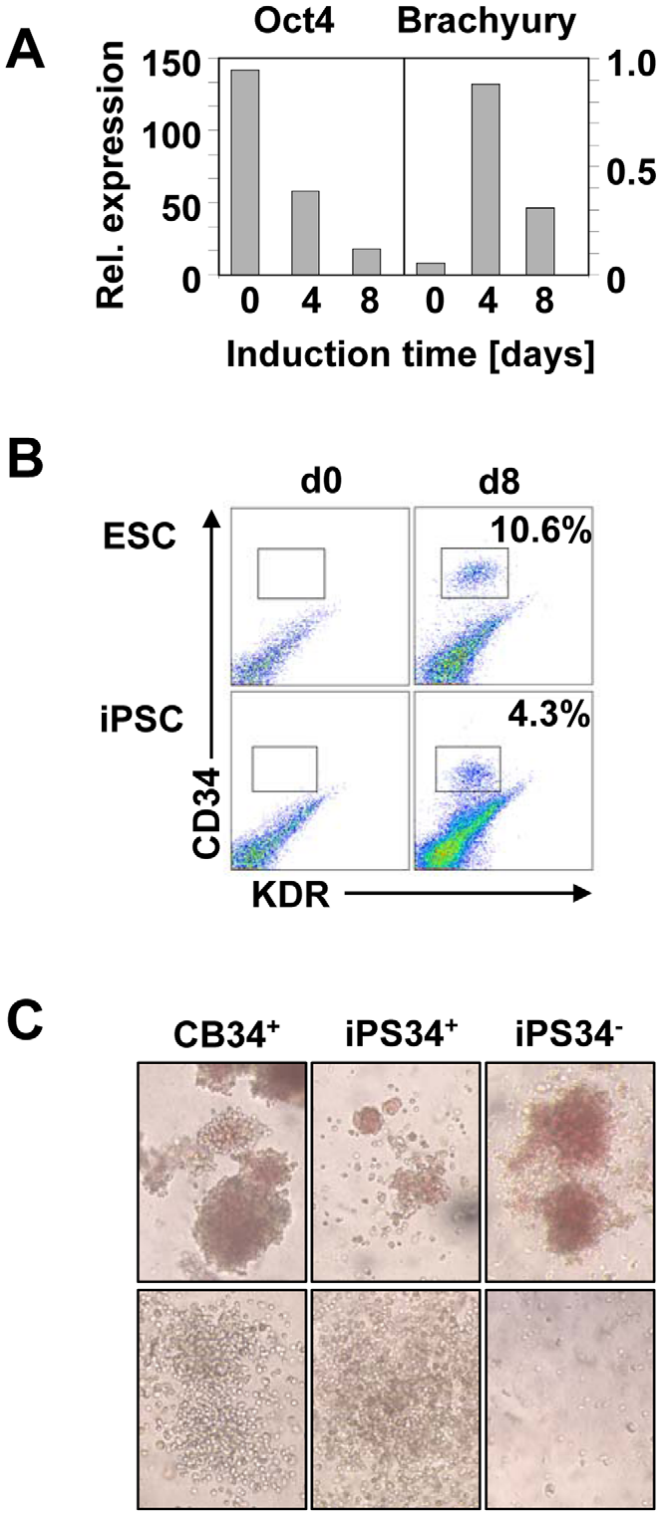
Hematopoietic differentiation of human iPSC. (A) Oct-4 and Brachyury mRNA expression were assessed by quantitative PCR at the onset of the differentiation (d0), and 4 (d4) and 8 (d8) days later. The expression of the Gus-B housekeeping gene was used for normalization (representative data of 3 experiments). (B) CD34 and KDR expression was assessed by flow cytometry. ESC are shown in the upper panels and iPSC in the lower panels. Expression prior to differentiation (d0, left panel) and after 8 days (d8, right panel) is depicted. (C) CFU morphology was assessed upon 14 days of methylcellulose culture of iPSC-derived cells. Erythroid CFUs (upper panels) and myeloid CFUs (lower panels) were observed in these cultures and CB-derived cells are shown (initial magnification ×100).

iPSC have recently been reported to recapitulate major ESC features, including the ability to differentiate in various cell lineages *in vitro* and to generate teratomas [12], [13], [14]. We therefore investigated whether iPSC generated in our laboratory upon reprogramming of HFF (see **Figure S1** for *in vitro* (**A-E**) and *in vivo* (**F-H**) characterization, and **Movie S1** for the presence of beating cardiomyocytes) behave like *bona fide* ESC, in particular when exposed to our protocol of hematopoietic induction. Indeed, as for ESC, iPSC rapidly down-modulated Oct-4 expression and transiently expressed Brachyury at day 4. This expression subsequently decreased when BMP-4 was substituted by early acting hematopoietic growth factors (**Fig. 3A**). This substitution was also associated with the emergence at day 8 of a CD34^+^-KDR^+^ population that was clearly distinguishable from its CD34^−^-KDR^−^ counterpart (**Fig. 3B**). Sorted iPS34^+^ cells displayed a similar phenotype as their ESC counterpart, including expression of CD31, CD43 and CXCR4 (**Fig 1C**, and data not shown). After sorting and culture in clonogenic conditions, both iPS34^+^ and iPS34^−^ produced hematopoietic colonies (**Fig. 3C**) with increased frequency of GM colonies in the iPS34+ fractions (**Table 1**). However, the large variability observed in these CFU experiments, including the absence of iPS34^−^ proliferation in many methylcellulose assays, did not allow establishing a significant difference of cloning efficiency between these 2 groups (**Table 1**). Nevertheless, the overall cloning frequencies (erythroid plus CFU-GM) were comparable to those of ESC and approximately 150-fold lower than CB34^+^ cell frequencies. Interestingly, iPS34^+^, as with ES34^+^, expressed ε and γ, but not β globins (not shown), consistent with a complete reprogramming of the initial fibroblasts to an embryonic stage. They also produced in some instances, GM-colonies of very large size that had not been observed in ES34^+^ cultures. Altogether, these data suggest that our protocol devoid of xenogenic compounds to generate hematopoietic cells could be successfully applied to reprogrammed somatic cells.

**Table 1:** Indicative frequencies of hematopoietic colonies recovered after clonogenic cultures of sorted iPSC.

	CB34^+^	iPSC 34^+^	iPSC 34^−^
**Erythroids**	4.7±2.1	0.041±0.052	0.029±0.013
**CFU-GM**	9.1±4.3	0.058±0.055	0.008±0.007
**Total**	14±6.4	0.099±0.110	0.037±0.020
**Ratio of responding cultures**	4/4	6/6	2/4

iPSC have been differentiated in six independent experiments, sorted into CD34^+^ and CD34^−^ fractions and tested in CFU assays. Frequencies are expressed as percentages of colonies recovered, compared to the initial cell seeding. Standard deviations are shown. The fraction of responding cultures are depicted on the last line. CFU: colony forming units, GM: granulocytes-macrophages. CB: cord blood-derived.

## Discussion

Our work describes the generation of hematopoietic progenitors from human ESC and iPSC induced by the sequential exposure to BMP-4 and bFGF [17], [19], and to a mixture of early-acting hematopoietic factors in the absence of any xenogenic compound and feeder cells. Several other protocols have reported the generation of hematopoietic progenitors from human ESC in fully defined serum- and feeder-free conditions [17], [19], and similarly to our differentiation procedure, these protocols initially rely on the ventralizing effect of BMP-4 to induce early hematopoietic commitment. Kennedy et al. further demonstrated that hematopoietic potential was strictly dependent on such signaling [17], however, the mixture of cytokines used subsequently, differed between these procedures. Pick et al. used BMP-4 simultaneously with VEGF, SCF and FGF2 for a minimum of 10 days, and reported 13.9% of CD34-expressing cells, with a mean CFC frequency of approximately 2.2×10^−3^. Kennedy et al report CFC potential in their KDR^+^/CD31^+^/CD34^+^ fraction at day 8 (about 3×10^−3^), however, they did not observe any CFC potential in their negative fraction. By supplementing our cultures with Flt3L, TPO and SCF from day 4 to day 8, we succeeded in increasing the potential of CD34 expressing cells (up to 20%) while achieving comparable CFC frequencies (about 2.5×10^−3^). Moreover, we observe a significant erythroid potential also from the CD34^−^ fraction, which resemble the sequential hematopoietic potential observed in protocols supplemented with serum and/or feeders [6]. We believe that this choice of early acting cytokines results in an overall differentiation that is more consistent to what observed *in vivo*.

For instance, culture supplementation with Flt3-L, which has not been documented before in such an animal protein-free context, may further enhance ESC and iPSC differentiation toward hematopoiesis *in vitro*. Indeed, Flt3-L is pivotal for the maintenance of CB34^+^ stem cells in long-term cultures [21], [22] and its serum level correlates with the hematopoietic requirement of the individuals. In particular, patients suffering from severe chronic hematopoietic dysfunctions such as Fanconi's anemia [23], [24] exhibit elevated serum levels of Flt3-L in response to the disease.

After 8 days of differentiation, our protocol produced ESC progeny comprising of CD34^+^ and CD34^−^ cells, both able to generate hematopoietic colonies in methylcellulose-based semi-solid cultures. ES34^+^ were CD45^−^ CD38^−^, KDR^+^ and CD31^+^, consistent with an immature hemangioblastic phenotype, though more recent studies claim that hemangioblasts, in addition to KDR, also express Brachyury [25] which is not the case here. Thus, our cells may have differentiated beyond the hemangioblast stage prior to clonal methylcellulose assays. This is consistent with their slight expression of CD43, which has been previously reported as an early marker to define hematopoietic progenitors in human ESC cultures [4]. In contrast with earlier studies [17], our ES34^+^ cells did not express CD117 (c-kit). A significant fraction of ES34^+^ cells expresses CXCR4, the receptor for stroma-derived factor (SDF)-1/CXCL-12, which is associated with the homing and maintenance of HSC in the bone marrow. This observation was not reported with either ESC (H1 cell line) or iPSC differentiated in presence of OP9 stroma [3], [4], and suggests that our protocol may achieve the generation of hematopoietic cells with enhanced *in vivo* bone marrow homing properties [26]. While this issue remains to be investigated *in vivo* using appropriate reconstitution models [27], we presently consider it unlikely that our cells, given their primitive-stage phenotype, would achieve a robust *in vivo* reconstitution. Although not in the primary intention of the present study, this point deserves future investigations.

Interestingly, the hematopoietic transcription factors SCL/Tal1 and Runx1 were both exclusively detectable in the CD34^+^ fraction. This observation is puzzling as SCL expression is associated with the short-lived, primitive hematopoiesis, whereas Runx1 is required for definitive hematopoiesis [20]. Thus, the expression of both markers in the same cell fraction appears rather contradictory. One can speculate that the *in vitro* environment may artificially block CD34^+^ cells at an intermediate stage between primitive and definitive hematopoiesis (that is not observed in unmanipulated cells) which may allow the expression of both transcripts in the same cells. Alternatively, we cannot exclude that cultures may contain a mixture of cells exclusively expressing either factor.

ES34^−^ cells were SCL^−^, Runx1^−^ and differentiated mostly in erythroids, suggesting that they were locked at a rather primitive stage of hematopoiesis. Indeed, the morphology of our erythroid colonies generated from ES34^−^ cells, as well as their inability to generate β-globin, are identical to those described by Kennedy et al. [17], further suggesting that ES34^−^ cells likely represent progenitors of primitive and not definitive hematopoiesis. The few macrophagic colonies observed in the ES34^−^ fraction may originate from either CD34^+^ contaminant cells that were too few to be detected by FACS, or from progenitors of primitive hematopoiesis that were endowed with the ability to produce GM colonies at a low frequency [17].

Despite the identification of a sizeable frequency of hematopoietic progenitors after clonogenic assays in methylcellulose, the overall efficacy of our protocol remains low, and for the time being, incompatible with eventual clinical application. Further improvements of the method are required to force ESC and iPSC toward definitive hematopoiesis. Foetal HSC may not be fully responsive to the hematopoietic growth factors present in the CFU assay, and such a lack of response may contribute to the apparent paucity of HSC generated *in vitro*. Indeed, we cannot discern whether the observed low frequency of progenitors (i.e. 100 fold less than in CB) is due to the real absence of these cells or is related to their inability, probably due to a lack of sufficient differentiation, to respond efficiently to the classical assay performed in this study. Moreover, in light of recently published data [28], the particularly low hematopoietic efficiency of iPSC may also be due to an increased sensitivity to apoptosis as compared to ESC *in vitro.*

Further investigations and new approaches are required to establish the full potential of ESC and iPSC once induced toward the hematopoietic lineage *in vitro*. Nevertheless, this work contributes to the demonstration that it is possible to generate *in vitro* hematopoietic progenitors from ESC and iPSC in an environment devoid of xenogenic compounds, which represent one of the requirements to be fulfilled before manipulated cells can be envisaged for use in clinical applications.

## Materials and Methods

### Human ESC and iPSC cultures

Human embryonic stem cell lines (H1 line [1]; HS181 [29] and HS401 [30]) and human induced pluripotent stem cells (iPSC-w line, referred in the text as iPSC, derived from lentivirally transduced human foreskin fibroblasts (HFF) with OCT-4/SOX2/NANOG/LIN28 reprogramming genes as initially demonstrated in [13]) were maintained and expanded over irradiated HFF [14]. Knock-out serum (KSR, Invitrogen)-supplemented media was used for propagation, supplemented with 4 ng/ml of basic fibroblast growth factor (bFGF, R&D Systems) for ESC cultures, and 100 ng/ml bFGF for iPSC cultures. Cells were fed on a daily basis and weekly passaged using collagenase IV (1 mg/ml, Roche) or manual dissection when required.

### Hematopoietic differentiation

Confluent human ESC and iPSC colonies were disrupted to small aggregates using collagenase IV (1 mg/ml, 15 minutes at 37 °C). Cell aggregates were resuspended in StemPro fully defined medium (StemPro-34 Serum-Free Media, Gibco proprietary formulation, guaranteed as animal protein-free by the manufacturer) supplemented with 0.5 ng/ml human recombinant BMP-4 (R&D Systems) at a ratio of 2 confluent ESC/iPSC wells for 1 well of differentiation. During all the differentiation process, cell attachment was prevented using low cluster tissue culture dishes (Corning Costar). On day 1 of differentiation, embryoid bodies (EB) were harvested and transferred to fresh StemPro media supplemented with 10 ng/ml BMP-4 and 5 ng/ml bFGF. After 72 hours, EB were harvested again and transferred to « hematopoietic medium » consisting of StemPro media supplemented with 100 ng/ml human recombinant VEGF (R&D Systems), 5 ng/ml bFGF, 100 ng/ml SCF (kind gift from Amgen), 100 ng/ml FLT3-L (kind gift from Amgen) and 40 ng/ml TPO (Peprotech, UK) for 4 additional days. All differentiation steps were performed in hypoxic conditions (5% O_2_) in a humidified incubator at 37 °C.

### Analysis of hematopoiesis and clonogenic assays

After 8 days of differentiation, EB were harvested and spun at 65 g for 4 min. Supernatants containing non-adherent cells were stored on ice and EB were disrupted using Trypsin-EDTA followed by manual disruption in FBS-containing blocking medium using a 21G needle. After centrifugation, cells were resuspended in freshly prepared collagenase IV solution (0.2 mg/ml) for 30 min at 37 °C. Cells were disrupted again using a 21G needle and filtered over a 70 µm cell strainer (Falcon). Supernatants from the initial centrifugation were pooled with these suspensions and magnetic bead associated cell sorting (MACS) for the CD34 epitope was performed following manufacturer's instructions (Miltenyi Biotech). Human clonogenic progenitor assays were performed by plating undifferentiated ESC, ESC-derived CD34^+^ and CD34^−^ cells as well as CB34^+^ cells in a methylcellulose-containing medium (Methocult GF H84434, Stem Cell Technologies France). All clonogenic progenitor assays were performed in duplicates. Counts were performed after incubation at 37 °C, 5% CO_2_ for 14 days in a humidified atmosphere. In some instances, cells from CFU assays were washed out from the methylcellulose by gentle pipetting in Hank's balanced solution. Single cell suspensions were utilized for flow cytometry analysis or RNA extraction.

### Flow cytometry

Cell suspensions were analyzed after staining with antibodies specific for CD34, CD45, CXCR4, KDR (VEGF-receptor), CD117, CD38, CD31, CD43, MHC class I, MHC class II, Glycophorin-A (GPA) (BD Biosciences, Miltenyi and R&D Systems). Dead cells were identified as 7-amino actinomycin D (7AAD)-high cells and excluded. Cells were stained in PBS containing 20% of human AB serum (Sigma) to prevent unspecific binding. Data were collected on a FACS Calibur device (BD Biosciences) and analyzed with the Flowjo software (Treestar).

### Reverse transcription, PCR and quantitative PCR analysis

Total RNA was extracted using the RNA easy Microkit (Qiagen) following manufacturer's instructions, including a DNAse I treatment to remove residual genomic DNA. cDNA was synthesized with random hexamers and Superscript II reverse transcriptase (Invitrogen). Real-time PCR was performed using a sequence detector (model ABI PRISM 7900HT; Perkin-Elmer). Primers for quantitative RT-PCR were: SCL/Tal1 forward GGAGACCTTCCCCCTATGAG, reverse GATGTGTGGGGATCAGCTTG (147 bp); Brachyury forward ACAGCTGTGACAGGTACCCAAC, reverse CATGCAGGTGAGTTGTCAGAAT (109 bp); Oct-4 forward AGTGCCCGAAACCCACACTG, reverse ACCACACTCGGACCACATCCT (81 bp); GusB forward CCACCAGGGACCATCCAAT, reverse AGTCAAAATATGTGTTCTGGACAAAGTAA (79 bp); GATA4: forward CTGGCCTGTCATCTCACTACG, reverse GGTCCGTGCAGGAATTTGAGG; Nxk2.5 forward CTATCCACGTGCCTACAGCGAC, reverse GCACAGCTCTTTCTTTTCGGC; and MEF2C forward TTCAACGCTGGACGAAGTAA, reverse CAGTTCCCAAATTCCTGCAT. For RT-PCR, globin and Runx1 primers were as described by Kennedy et al. [17] and Vodyanik et al. [3] respectively, while pluripotency markers were as follows: Oct-4 forward GACAACAATGAAAATCTTCAGGAGA, reverse TTCTGGCGCCGGTTACAGAACCA; Nanog forward AAGACAAGGTCCCGGTCAAG, reverse CCTAGTGGTCTGCTGTATTAC; Sox2 forward GCGAACCATCTCTGTGGTCT, reverse GGAAAGTTGGGATCGAACAA, Rex1 forward CCTAGTGGTCTGCTGTATTAC, reverse CCTAGTGGTCTGCTGTATTAC; GAPDH forward AGCCACATCGCTCAGACACC, reverse GTACTCAGCGGCCAGCATCG.

## Supporting Information

Figure S1 In vitro and in vivo characterization of the iPSC (w line). (A) Phase contrast picture of undifferentiated iPSC colonies on irradiated HFF; (B) expression of pluripotency markers by RT-PCR, H1 ESC are used as positive controls; (C) immunofluorescence staining of the pluripotency markers Oct-4, Nanog, Tra-1-81, SSEA4 and Tra1-60 (C). Feeder cells appear in green because stably expressing GFP as previously described [14]. (D) Example of in vitro cardiac differentiation through EB formation and (E) expression levels by Q-PCR to three cardiac transcription factors at indicated time points. Black bars represent iPSC-W and grey bars, H1-ESC positive controls. (G-I) Cytochemical analysis (H&E staining) of in vivo teratoma formation shows tissues of the three germ layers.
(4.20 MB TIF)

Movie S1 Beating cardiomyocyte cluster from differentiating iPSC-derived embryoid bodies.
(1.31 MB AVI)
