# Supplementary figures and images for: Human Pluripotent Stem Cells Differentiated in Fully Defined Medium Generate Hematopoietic CD34+ and CD34− Progenitors with Distinct Characteristics
Source: PLoS One. 2011 Feb 25;6(2):e14733. doi: 10.1371/journal.pone.0014733 (PMC3045374; doi:10.1371/journal.pone.0014733)

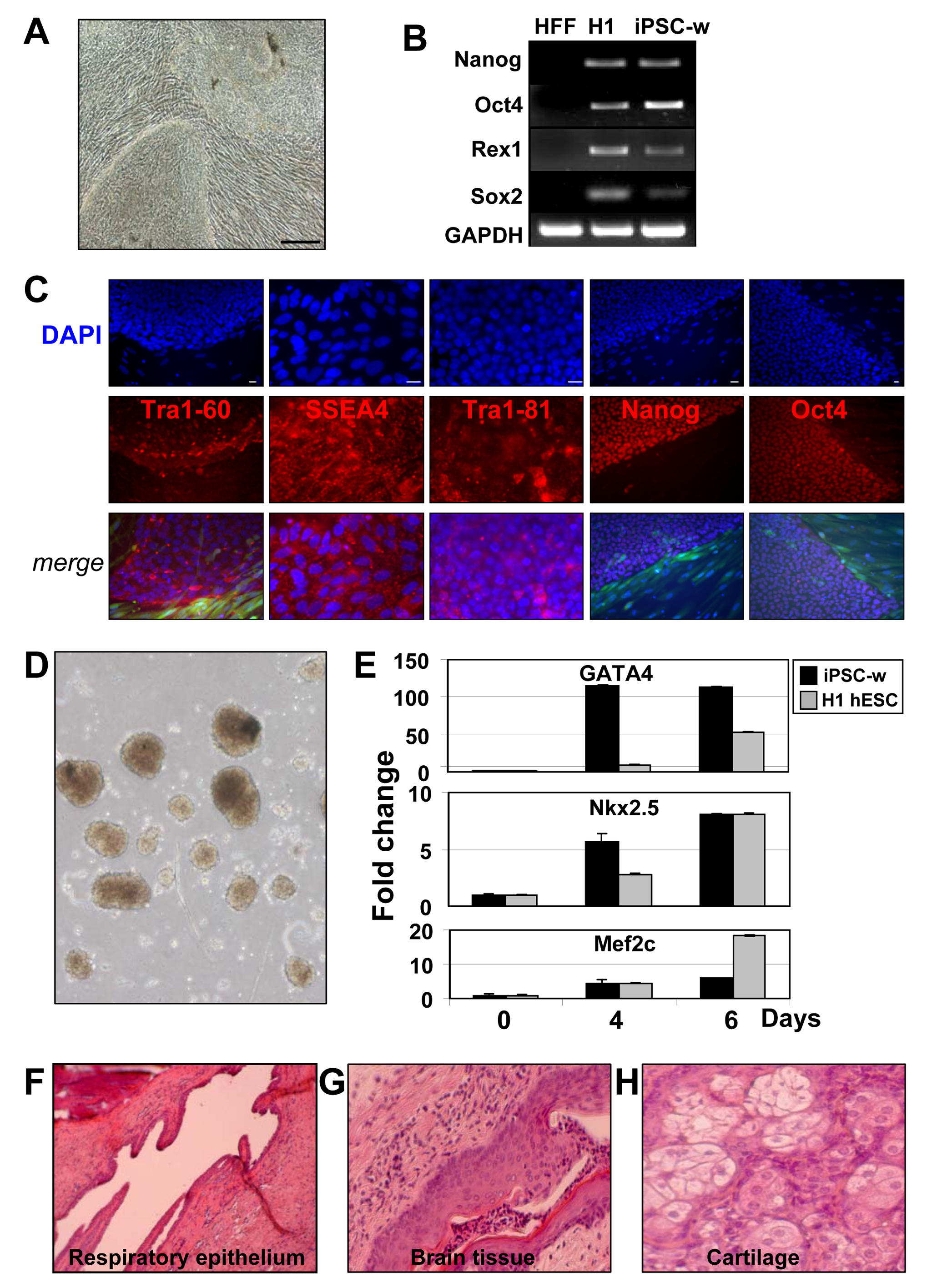

Supplement: Figure S1 — In vitro and in vivo characterization of the iPSC (w line). (A) Phase contrast picture of undifferentiated iPSC colonies on irradiated HFF; (B) expression of pluripotency markers by RT-PCR, H1 ESC are used as positive controls; (C) immunofluorescence staining of the pluripotency markers Oct-4, Nanog, Tra-1-81, SSEA4 and Tra1-60 (C). Feeder cells appear in green because stably expressing GFP as previously described [14]. (D) Example of in vitro cardiac differentiation through EB formation and (E) expression levels by Q-PCR to three cardiac transcription factors at indicated time points. Black bars represent iPSC-W and grey bars, H1-ESC positive controls. (G-I) Cytochemical analysis (H&E staining) of in vivo teratoma formation shows tissues of the three germ layers. (4.20 MB TIF) [file pone.0014733.s001.tif]
